# The asthma candidate gene *NPSR1 *mediates isoform specific downstream signalling

**DOI:** 10.1186/1471-2466-11-39

**Published:** 2011-06-27

**Authors:** Christina Orsmark Pietras, Johanna Vendelin, Francesca Anedda, Sara Bruce, Mikael Adner, Lilli Sundman, Ville Pulkkinen, Harri Alenius, Mauro D'Amato, Cilla Söderhäll, Juha Kere

**Affiliations:** 1 Department of Biosciences and Nutrition, Karolinska Institutet, Stockholm, Sweden; 2 Department of Medical Genetics, University of Helsinki and Folkhälsan Institute of Genetics, Helsinki, Finland; 3 Istituto di Neurogenetica e Neurofarmacologica, Consiglio Nazionale della Ricerche, Monserrato, Italy; 4 The National Institute of Environmental Medicine, Division of Physiology, Karolinska Institutet, Stockholm, Sweden; 5 Unit of Excellence for Immunotoxicology, Finnish Institute of Occupational Health, Helsinki, Finland; 6 Science for Life Laboratory, Stockholm, Sweden

## Abstract

**Background:**

Neuropeptide S Receptor 1 (*NPSR1, GPRA, GPR154*) was first identified as an asthma candidate gene through positional cloning and has since been replicated as an asthma and allergy susceptibility gene in several independent association studies. In humans, *NPSR1 *encodes two G protein-coupled receptor variants, NPSR1-A and NPSR1-B, with unique intracellular C-termini. Both isoforms show distinct expression pattern in asthmatic airways. Although NPSR1-A has been extensively studied, functional differences and properties of NPSR1-B have not yet been clearly examined. Our objective was to investigate downstream signalling properties of NPSR1-B and functional differences between NPSR1-A and NPSR1-B.

**Methods:**

HEK-293 cells transiently overexpressing NPSR1-A or NPSR1-B were stimulated with the ligand neuropeptide S (NPS) and downstream signalling effects were monitored by genome-scale affymetrix expression-arrays. The results were verified by NPS concentration-response and time series analysis using qRT-PCR, cAMP and Ca^2+ ^assays, and cAMP/PKA, MAPK/JNK and MAPK/ERK pathway specific reporter assays.

**Results:**

NPSR1-B signalled through the same pathways and regulated the same genes as NPSR1-A, but NPSR1-B yielded lower induction on effector genes than NPSR1-A, with one notable exception, CD69, a marker of regulatory T cells.

**Conclusions:**

We conclude that NPSR1-B is regulating essentially identical set of genes as NPSR1-A, with few, but possibly important exceptions, and that NPSR1-A induces stronger signalling effects than NPSR1-B. Our findings suggest an isoform-specific link to pathogenetic processes in asthma and allergy.

## Background

Neuropeptide S receptor 1 (*NPSR1 *also *GPRA, GPR154*) was first identified as an asthma susceptibility gene through positional cloning [1]. The genetic evidence was supported by significant single nucleotide polymorphism (SNP) and haplotype associations to asthma in three separate populations. To date, the association of NPSR1 to asthma and allergy has been replicated in seven independent populations [2-8]. Studies have also reported involvement of *NPSR1 *in inflammatory disorders of skin and intestine [9,10], neurally related traits such as sleep and circadian phenotypes [11] and anxiety [12].

**Figure 1:**
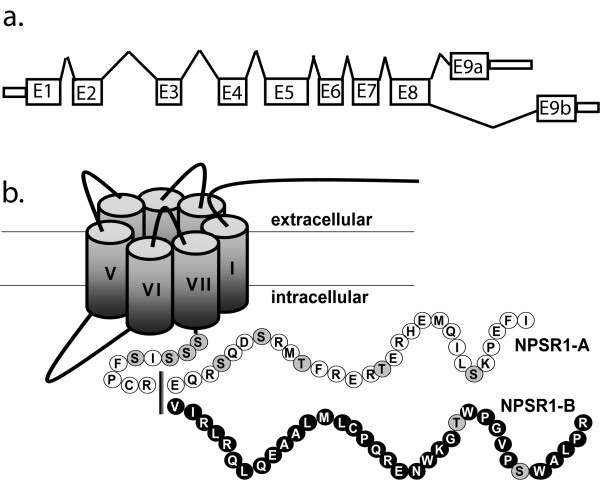
**Schematic picture showing the exonic structure (a) and the isoforms (b) of *NPSR1-A *and *NPSR1-B***. *NPSR1-A *encodes the shorter protein isoform with a 29 aa long distinct C-terminus. *NPSR1-B *uses an alternate 3' exon (E9b) in encoding the larger protein with a 35 aa long distinct C-termini. Shadowed circles in the C-termini (b) represent putative phosphorylation sites (S = serine, T = threonine).

NPSR1 is a 7-transmembrane G-protein coupled receptor (GPCR) phylogenetically related to other neuropeptide receptors such as neuropeptide Y (NPY), neurotensin and tachykinin receptors [13]. Upon stimulation by neuropeptide S (NPS), the natural ligand for NPSR1, downstream signalling has been shown to be mediated through intracellular coupling to Gαq and Gαs [14,15]. Several *NPSR1 *splice variants have been identified but only two, NPSR1-A and NPSR1-B are effectively transported to the plasma membrane [16]. These two full-length splice variants differ in their 3' ends possessing alternate terminal exons 9a or 9b (Figure 1a) which encodes distinct carboxy-terminal peptide chains (Figure 1b). The C-terminus is important for many stages of a GPCR protein lifespan and modifications can affect, e.g., transportation to the cell membrane, anchoring and downstream signalling [17]. Upon receptor activation, conformational changes reveal phosphorylation sites on the C-terminus. These sites are phosphorylated by G protein coupled receptor kinases (GRKs) and can bind GPCR activity regulating proteins, arrestins. Upon arrestin binding a receptor is targeted for internalization and recycling, redirected to G-protein independent signalling pathways such as mitogen-activated protein kinase (MAPK) pathway, or degraded [18].

The regulatory mechanisms of alternative splicing are not yet fully understood for NPSR1. However, it is clear that NPSR1 isoforms demonstrate distinct expression pattern in tissues and cells, with NPSR1-A generally showing a more ubiquitous expression, and that both isoforms have specific roles in inflammation, as previously shown for NPSR1-B, which is up regulated in asthmatic airways [1,9,10,16,19]. High total NPSR1 expression is found in murine brain [20] and it has been suggested that NPSR1 might contribute to an inflammatory phenotype by neurally mediated mechanisms [21,22]. It has been shown that the receptor potency for NPSR1 is dependent on the Ile107Asn isoform, where the NPSR1 Ile107 isoform gives a greater downstream response upon NPS stimulation than 107Asn [23-25]. In our study the NPSR1-A and -B constructs contain the more potent Ile107 isoform.

Even though these data suggest importance of both receptor signalling pathways, little is known about any functional disparity between these isoforms. In this study, we aimed to identify signal properties of NPSR1-B and clarify differences of downstream signalling between the NPSR1-A and NPSR1-B isoforms.

## Methods

### Cell culture

Human embryonic kidney cells (HEK-293) were cultured in MEM+GlutaMAX-1 medium (Gibco/Invitrogen) supplemented with 10% fetal calf serum (FCS) (Biosera), 1% sodium pyruvate, 1% non-essential amino acids and 1% penicillin/streptomycin (Gibco/Invitrogen). Human epithelial lung carcinoma cells (A549) were cultured in F-12/D-MEM medium (1:1; Gibco/Invitrogen) supplemented with 10% FCS, L-glutamine and 1% penicillin/streptomycin. Human neuroblastoma cells (SH-SY5Y) were cultured in MEM + GlutaMAX-1 medium supplemented with 10% FCS and 1% penicillin/streptomycin. All cells were kept at 37°C in a humified 5% CO_2 _incubator. The cells were transiently transfected (Lipofectamin™2000 Reagent, Invitrogen, Carlsbad, USA) with equal amounts of pCMV-*NPSR1-A*,- *NPSR1-B *(construction of the expression vectors has been described earlier) [16] or an empty pCMV vector control in a ratio of 1:2 (DNA:Lipofectamin). 24 h post transfection cells were stimulated with NPS (SFRNGVGTGMKKTSFQRAKS) (Sigma-Genosys LTD, Haverhill, UK or New England Peptide™, Gardner, MA, USA).

### RNA isolation and cDNA synthesis and quantitative real-time PCR (qRT-PCR)

**Figure 2:**
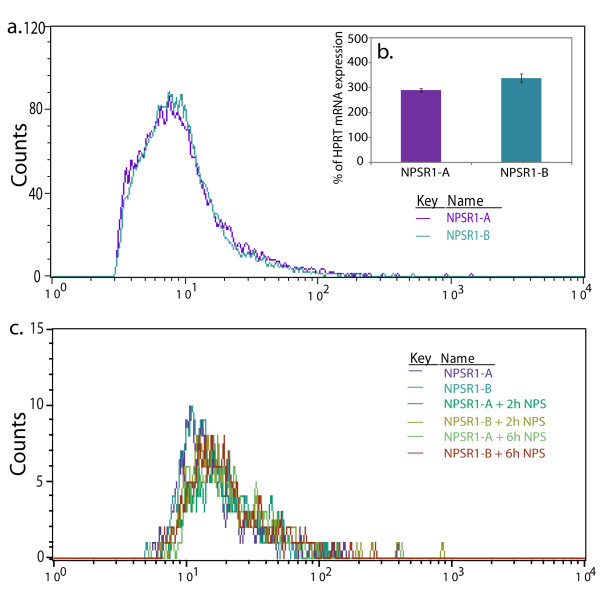
**NPSR1 protein and mRNA expression in transient transfected HEK-293 cells**. Transiently NPSR1-A or NPSR1-B overexpressing HEK-293 cells, with or without NPS stimulation, were stained with an N-terminal monoclonal antibody together with a FITC conjugated secondary antibody, and the fluorescence intensity was measured. The histogram plot (a) shows that the intensity, representing the amount of receptors expressed on the cell membrane, and the transfection efficiency, represented by the number of counts, is the same between the two isoforms. From the same experiment NPSR1 mRNA expression was measured showing that the level of NPSR1-A and -B mRNA is similar between the respective overexpressing cells as well (b). Histogram plot (c) illustrates the intensity of the two isoforms after 2 and 6 hours of NPS stimulation.

Total cellular RNA was isolated with the RNAeasy Mini Kit (Qiagen, Hilden, Germany) and reverse transcription was done using the iScript™ cDNA synthesis kit (Bio-Rad Laboratories, Hercules, CA, USA) or SuperScript™ First-Strand Synthesis System (Invitrogen, Carlsbad, USA) according to the manufacturer's protocol. All experiments were performed in biological duplicates or triplicates. qRT-PCR was performed using Fast SYBR^® ^Green technology with hypoxanthine-guanine phosphoribosyl transferase (HPRT) or glyceraldehyde-3-phosphate dehydrogenase (GAPDH) as endogenous controls. Gene-specific primers were designed using Primer3 software (for complete lists of all primer sequences please see Additional file 1, Table S1). The PCR assays were performed in a total volume of 10 μl, containing 2 ul cDNA template, 5 ul Fast SYBR^® ^Green PCR Master Mix (Applied Biosystems, Foster City, CA, USA) and 100 nM of each primer. A 7500 Fast Real-Time PCR system (Applied Biosystems) was used according to manufacturer's instructions. A dissociation stage was added to confirm primer specificity. All assays were carried out in technical duplicates or triplicates. Relative quantification and calculation of the range of confidence was performed with the comparative ΔΔCT method [26]. Results are shown as relative expression compared to NPS stimulated empty vector control cells with error bars illustrating the standard error of the mean (SEM). The levels of total NPSR1 mRNA and isoform specific NPSR1-A and NPSR1-B mRNA were measured with qRT-PCR to control for equal isoform expression levels prior to any experiment on all transiently transfected cells (representative data shown Figure 2b).

### Fluorescence based quantification

24 h after transfection transiently NPSR1-A or -B overexpressing HEK-293 cells, stimulated with 1 μM NPS or left untreated, were incubated 15 min in room temperature with mouse monoclonal antibodies specific for the N-terminal NPSR1 peptide sequence TEGSFDSSGTGQTLDSSPVA (Quattromed, Tartu, Estonia). The incubated cells were washed with PBS+10% cell dissociation buffer, Hanks'-based (Invitrogen, Carlsbad, USA) followed by 15 min incubation of a FITC conjugated goat antimouse secondary antibody (Abcam, Cambridge, UK). The NPSR1 expression on the plasma membrane was analysed with BD FACSCalibur™. Data analysis was performed with the CellQuestPro software. Background correction was made for HEK-293 cells incubated with secondary antibody only. Prior to antibody incubation an aliquote of the cells were removed and lysed for NPSR1 RNA expression analysis.

### Expression-array sample preparation

For the expression-array experiment, *NPSR1-A*, -*B *or an empty pCMV vector was transiently transfected into HEK-293 cells and stimulated for 6 h with 2 μM NPS. Total RNA (see section above) from each sample was used for cDNA synthesis according to the Affymetrix protocol. A total of 6 hybridizations (biological duplicates) and scannings (Affymetrix GeneChip Scanner 3000) were carried out using standard Affymetrix protocols for gene expression http://www.affymetrix.com with the HGU133plus2 array.

### Expression-array data analyses

Experimental data were quality assessed, pre-processed, and analyzed as previously described [27] using the statistical software R http://www.R-project.org by implementing the packages Affy, limma, HGU133plus2 and kth [28-30]. A B-test (empirical Bayes shrinkage) was used to extract log-odds of differential expression (B-values). The B-statistics has an empirical distribution and cut-offs for differential expression have to be tailored for each experiment. B-value >7 was used as cut-off to derive a list of prioritized genes. The expression data is available at http://www.ebi.ac.uk/arrayexpress/ with the ArrayExpress accession: E-MEXP-2782.

### NPS concentration-response and time series

To refine concentration and time dependency transiently NPSR1-A, -B or empty pCMV vector overexpressing cells were treated either with 0.001, 0.01, 0.1, 1 or 10 μM NPS for 6 h (concentration-response) or with 2 μM NPS for 30 min, 1, 2, 4, 6, 12, 24 or 48 h (time-response). To verify the results in other cell types, A549 and SH-SY5Y transiently overexpressing NPSR1-A, -B or an empty pCMV vector were treated with 2 μM NPS for 6 h. mRNA expression was measured with qRT-PCR. Agonist concentration-effect curve data were fitted to the Hill equation using an iterative, least squares method (GraphPad Prism, San Diego, CA, U.S.A), to provide estimate EC_50 _(the agonist concentration that induces half-maximum effect) and presented as -log_10 _(pEC_50_) which is normally distributed.

### Measurements of intracellular cAMP accumulation and Ca^2+ ^mobilization

To assay cAMP accumulation, transiently NPSR1-A or -B overexpressing HEK-293 cells were 24 h after transfection incubated for 40 min with 200 μM 3-isobutyl-1-methylxanthine (Sigma-Aldrich, St. Louis, MO, USA), a nonspecific inhibitor of phosphodiesterases [31]. The cells were then stimulated with 0.001, 0.01, 0.1, 1 or 10 μM NPS for 20 min, or left untreated, and prepared and stored at -80°C until assayed in six replicas by the enzyme immunoassay (EIA; 581001, Cayman Chemical, Ann Arbor, MI, USA) according to the manufacturer's instructions. For the Ca^2 ^assay, cells were seeded into 96-well plates, transiently transfected with NPSR1-A or -B and 24 h post-transfection the Fluo-4 NW calcium assay kit was used (F36205, Molecular Probes, Eugene, OR, USA) according to the manufacturer's instructions. The fluorescence intensity was measured before and after injection of 1 μM NPS using the Fluostar Optima microplate reader (BMG Labtechnologies, Offenburg, Germany).

### Luciferase reporter assay

Firefly-Luciferase reporter vectors for cAMP/PKA, MAPK/JNK and MAPK/ERK pathways were purchased from SABiosciences (Frederick, MD, USA), and used according to the manufacturer's instructions. Briefly, HEK-293 cells were transiently co-transfected with one of the above reporter vectors, a renilla-luciferase reporter vector for normalization (pRL-TK) and either an empty pCMV vector, *NPSR1-A *or -*NPSR1-B*. Cells were stimulated for 6 hours with 1 μM NPS (or left untreated). Luciferase assays were carried out with the Dual-Luciferase^® ^Reporter Assay System (Promega, Madison, WI, USA) according to manufacturer's instructions. Firefly luciferase activities were normalized to the values obtained for renilla luciferase, and expressed as fold induction relative to cells unstimulated and transfected with the empty vector.

### Phosphorylation site-directed mutagenesis

Phosphorylation site directed mutagenesis was performed on previously described pCMV-*NPSR1-A *and -*NPSR1-B *constructs with the QuickChange^® ^II-E site-directed mutagenesis kit (Stratagene, La Jolla, CA, USA). Primers for each respective mutagenesis were designed using Stratagene's web-based QuickChange^® ^primer design program http://www.stratagene.com/qcprimerdesigne (primer sequences are found in Additional file 1; Table S1). When designing the mutant constructs serine (S) and threonine (T) residues were exchanged for alanine (A) residues as follows: for the NPSR1-A unique C-terminal; all three S sites and two T sites were exchanged for A (AΔ5p), the two most N-terminal S residues and the most N-terminal T residue (AΔ3p) and the most C-terminal T and S residue (AΔ2p), for the NPSR1-B unique C-terminal; the only S and T residue on the -B isoform-tail were exchanged for A (BΔ2p) (for a schematic picture of the modified C-termini please see Additional file 2, Figure S1). The modified inserts of all clones were sequence-verified. HEK-293 cells were transient transfected and stimulated with 2 μM NPS for 1, 6 and 24 h, gene expression was monitored using qRT-PCR as described above.

### Statistical analysis

Data are expressed as mean ± SEM, unless stated otherwise. Student's *t*-test was used for parametric comparison between NPSR1-A and NPSR1-B, and p ≤ 0.05 was regarded as significant

## Results

### Protein and mRNA expression of NPSR1 overexpressing cells

Human embryonic kidney cells (HEK-293) express low levels of endogenous NPSR1 [27]. To assure that the NPSR1-A or -B transiently transfected HEK-293 cells expressed the isoforms in equal amounts, we measured the intensity of receptor protein on the plasma membrane with fluorescence based quantification, and mRNA with qRT-PCR (Figure 2). The FACS analysis showed that the transfection efficiency between the two isoforms was identical, with equal number of positive cell counts, and that the intensity of antibody bound receptor on each cell was similar both before and after NPS stimulation (Figure 2a, c). The mRNA levels between the NPSR1-A or NPSR1-B overexpressing cells was also comparable and in concordance with the protein expression (Figure 2b). The receptors mRNA expression levels were stable in the transient transfected cells up to seventy-two hours post-transfection (data not shown).

### Genome-scale affymetrix expression-arrays

**Figure 3:**
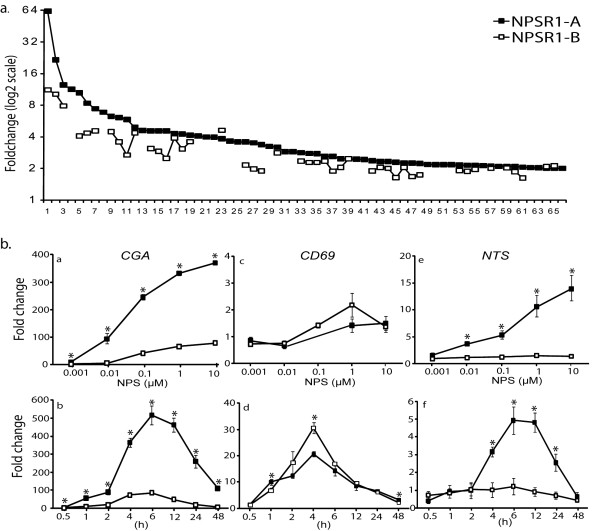
**Gene expression measured downstream NPS stimulation of transiently NPSR1-A or NPSR1-B overexpressing HEK-293 cells**. (a) NPSR1-A and -B differentially expressed genes as assessed using affymetrix expression-arrays revealed 66 genes induced by the -A isoform (cut off = ≥2 fold), of which 44 were induced by -B. No unique genes were induced by the -B isoforms. Genes are sorted by fold change, starting with the most differentially expressed gene as 1 (the numbers on the x-axis correspond to the order of the genes depicted in table 1). (b) qRT-PCR verification showing relative expression of *CGA, CD69 *and *NTS *after NPS-NPSR1 concentration- and time-response experiments. *CGA*, representing the majority of the genes in the expression-array experiment, demonstrate NPSR1-A as an isoform that induces stronger signalling effects both for NPS concentration-response and time-response. *CD69 *was the only gene greater induced by NPSR1-B and *NTS *did not respond to NPSR1-B signalling. Data shown as means relative to an NPS stimulated empty vector control ± SEM. * Indicates at which concentrations or time points the difference between NPSR1-A and -B was significant (p ≤ 0.05).

To investigate potential signalling differences between the two NPSR1 isoforms, we transiently transfected HEK-293 with *NPSR1-A *or *NPSR1-B*. We stimulated transfected cells and controls with NPS and monitored downstream signalling effects using the genome-scale Affymetrix HGU133plus2 expression-arrays. Biological duplicate samples were assayed and differentially regulated genes were identified by comparing NPS stimulated NPSR1-A or -B transfected cells against NPS stimulated pCMV-vector transfected cells. A stringent statistical threshold was used to derive a list of regulated genes (B > 7), prioritized based on likelihood of differential expression. Using this criterion, a total of 111 genes were identified as differentially expressed by NPSR1-A signalling (109 up regulated and two down regulated) and 55 genes by NPSR1-B signalling (all up regulated). 56 genes were found to be NPSR1-A specific but no genes were NPSR1-B specific. The 30 most differentially regulated genes with a more than three-fold up regulation by the NPSR1-A isoform and the corresponding fold change values for NPSR1-B are listed in Table 1. The most highly induced gene, *CGA*, was up regulated five times more by NPSR1-A compared to NPSR1-B and only one gene, *CD69*, was more induced by the -B isoform. Overall, the expression-array data suggested that NPS stimulation of NPSR1-A and -B isoforms up regulated the same genes but that NPSR1-A consistently yielded a higher induction of genes (Figure 3a).

**Table 1:** The >3 fold change differentially expressed genes in the NPS-NPSR1-A and -B expression-array experiment.

*	Gene Symbol	Gene name	NPS-NPSR1-A *Vs *NPS-empty vector	**NPS-NPSR1-B*Vs *NPS-empty vector**
1	*CGA*	GLYCOPROTEIN HORMONES. ALPHA POLYPEPTIDE	63.0	11.2
2	*CCL20*	CHEMOKINE (C-C MOTIF) LIGAND 20	21.7	10.2
3	*IL8*	INTERLEUKIN 8	12.5	7.8
4	*PCK1*	PHOSPHOENOLPYRUVATE CARBOXYKINASE 1 (SOLUBLE)	11.4	-
5	*NR4A2*	NUCLEAR RECEPTOR SUBFAMILY 4. GROUP A. MEMBER 2	10.5	4.1
6	*NR4A3*	NUCLEAR RECEPTOR SUBFAMILY 4. GROUP A. MEMBER 3	8.4	4.4
7	*C8orf4*	CHROMOSOME 8 OPEN READING FRAME 4	7.4	4.6
8	*SERPINB2*	SERPIN PEPTIDASE INHIBITOR. CLADE B (OVALBUMIN). MEMBER 2	6.9	-
9	*CTGF*	CONNECTIVE TISSUE GROWTH FACTOR	6.3	4.5
10	*CXCL2*	CHEMOKINE (C-X-C MOTIF) LIGAND 2	6.1	3.6
11	*TAC1*	TACHYKININ. PRECURSOR 1	5.9	2.7
12	*EGR3*	EARLY GROWTH RESPONSE 3	4.9	4.4
13	*AREG*	AMPHIREGULIN (SCHWANNOMA-DERIVED GROWTH FACTOR)	4.6	-
14	*FOSB*	FBJ MURINE OSTEOSARCOMA VIRAL ONCOGENE HOMOLOG B	4.6	3.1
15	*CYR61*	CYSTEINE-RICH. ANGIOGENIC INDUCER. 61	4.6	2.9
16	*INHBA*	INHIBIN. BETA A (ACTIVIN A. ACTIVIN AB ALPHA POLYPEPTIDE)	4.6	2.5
17	*EGR1*	EARLY GROWTH RESPONSE 1	4.3	3.9
18	*FOS*	V-FOS FBJ MURINE OSTEOSARCOMA VIRAL ONCOGENE HOMOLOG	4.3	3.1
19	*MAFF*	V-MAF MUSCULOAPONEUROTIC FIBROSARCOMA ONCOGENE HOMOLOG F (AVIAN)	4.2	3.6
20	*NTS*	NEUROTENSIN	4.1	-
21	*TM4SF1*	TRANSMEMBRANE 4 L SIX FAMILY MEMBER 1	4.0	-
22	*SNAP25*	SYNAPTOSOMAL-ASSOCIATED PROTEIN. 25 KDA	4.0	-
23	*CD69*	CD69 ANTIGEN (P60. EARLY T-CELL ACTIVATION ANTIGEN)	3.8	4.6
24	*MMP10*	MATRIX METALLOPEPTIDASE 10 (STROMELYSIN 2)	3.6	-
25	*STC1*	STANNIOCALCIN 1	3.6	-
26	*ELL2*	ELONGATION FACTOR. RNA POLYMERASE II. 2	3.6	2.2
27	*LOC387763*	HYPOTHETICAL LOC387763	3.5	2.0
28	*GEM*	GTP BINDING PROTEIN OVEREXPRESSED IN SKELETAL MUSCLE	3.4	1.9
29	*BIRC3*	BACULOVIRAL IAP REPEAT-CONTAINING 3	3.3	-
30	*PTX3*	PENTRAXIN-RELATED GENE. RAPIDLY INDUCED BY IL-1 BETA	3.2	2.8

* Numbers corresponds to position in Figure 3.

### NPS concentration-response and time series analysis

To verify the results from the expression-arrays and to investigate if the difference between NPSR1-A and -B signalling might depend on NPS concentration or dynamics, we performed concentration- and time-response experiments (Figure 3b). HEK-293 cells were transiently transfected with NPSR1-A, -B or an empty pCMV-vector control and stimulated with either a fixed concentration of NPS (2 μM) for 0.5 - 48 h or stimulated with a dilution series of NPS (1 nM-10 μM) for a fixed time of 6 h. mRNA expression was measured by qRT-PCR for 11 genes from the expression-array experiment selected as follows; three genes with the highest relative fold change (*CGA*, *CCL20 *and *IL8*), four genes only induced by NPSR1-A (*PCK1*, *SERPINB2*, *AREG *and *NTS*), transcription factors (*NR4A2*, *EGR1 *and *FOS*), and *CD69 *as the only gene more induced by NPSR1-B in the expression-array experiments.

The concentration-response experiments showed that overall, both receptor isoforms responded to increased NPS concentrations with a greater induction of genes, but with the NPSR1-A isoform consistently mediating a stronger response. However, stimulation of NPSR1-B mediated a stronger concentration-response on *CD69 *and no concentration-response on *NTS*. The EC_50_-values that could be estimated for the effect of NPS on NPSR1-A (all genes apart from *CD69*) and on NPSR1-B (*NR4A2*, *CGA*, *AREG*, *SERPINB2*, *CCL20 *and *PCK1*) was generally close to 0.1 μM with the exception of the effect on *NR4A2 *which was approximately 10 nM for NPSR1-A (Table 2). In the NPS-NPSR1 time series study, genes could be categorised into early (*PCK1, NR4A2, EGR1 *and *FOS*), intermediate (*CGA, AREG*, *NTS *and *CD69*) and late (*CCL20, IL8, SERPINB2*) induced genes. The early induced genes consisted mainly of transcription factors. Upon induction of these genes, NPSR1-A mediated a stronger response than -B at the early peak of expression (0.5-1 h post NPS stimulation) while at later time points the relative expression difference diminished and was similar to NPSR1-B induction of the same genes. The intermediate induced genes all peaked in expression 4-6 h post NPS stimulation. The relative expression for NPSR1-B induced genes was overall lower than -A, with one exception, *CD69 *and no expression was seen over time for *NTS*. The late induced genes peaked in relative expression 12-24 h post NPS stimulation and the response was always greater if induced by NPSR1-A. *PCK1*, *SERPINB2 *and *AREG*, which were exclusively up regulated upon NPSR1-A stimulation in the gene expression array experiment, were induced also by NPSR1-B in the time and concentration series experiment, although with low relative expression. The results verified and refined the expression-array data by demonstrating that most genes were induced by both isoforms but greater induction was obtained by NPSR1-A, as represented by *CGA *(Figure 3b: a, b). As exceptions *NTS *(Figure 3b: e, f) was not induced and *CD69 *(Figure 3b: c, d) was more strongly induced by NPSR1-B (data for the remaining genes tested are shown in Additional file 3, Figure S2).

**Table 2:** NPS concentration-response curve analysis on NPSR1-A or -B overexpressing HEK-293 cells.

pEC_50 _values	NPSR1-A	NPSR1-B
*NR4A2*	8.10 ± 0.06	7.08 ± 0.27
*EGR1*	7.14 ± 0.27	n.d.
*FOS*	7.27 ± 0.21	n.d.
*CGA*	7.34 ± 0.05	7.00 ± 0.06
*AREG*	6.63 ± 0.31	6.77 ± 0.83
*NTS*	7.06 ± 0.23	n.d.
*IL8*	7.35 ± 0.14	n.d.
*SERPINB2*	7.62 ± 2.93	6.90 ± 0.61
*CCL20*	6.79 ± 1.67	7.00 ± 0.08
*CD69*	n.d.	n.d.
*PCK1*	6.79 ± 0.07	7.05 ± 0.15
		
cAMP	8.35 ± 0.17	8.41 ± 0.28

The EC_50 _value (the agonist concentration that induces half-maximum effect) is presented as -log_10 _(pEC_50_). Data are expressed as mean ± SEM. When a concentration-response curve not fitted to the Hill equation the pEC_50 _was not determined (n.d.).

**Figure 4:**
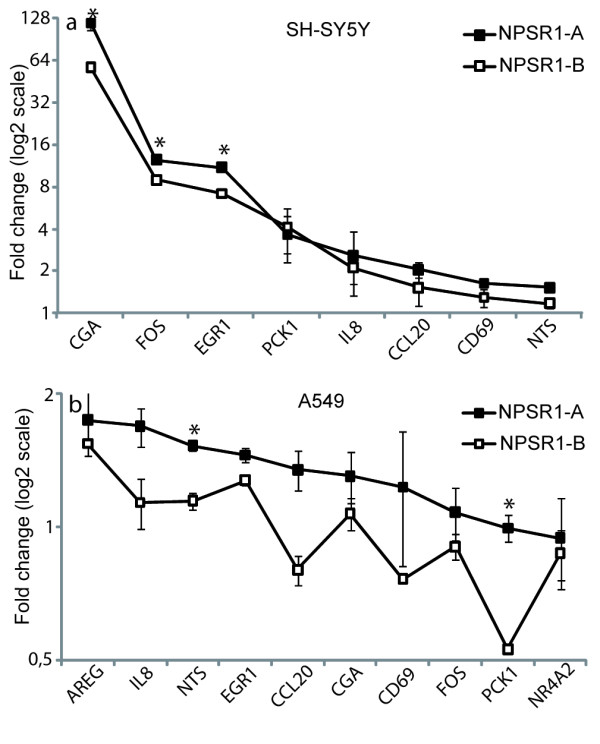
**Gene expression downstream transiently NPSR1-A or NPSR1-B overexpressing SH-SY5Y cells and A549 cells**. Relative mRNA expression was measured with qRT-PCR on NPS stimulated NPSR1-A or -B overexpressing SH-SY5Y cells (a) and A549 cells (b) for a subset of genes. NPSR1-A induced genes consistently showed a higher differential expression than NPSR1-B induced genes. Data shown as means relative to an NPS stimulated empty vector control ± SEM. * Indicates which genes that are significantly (p ≤ 0.05) different between NPSR1-A and -B.

To investigate if differences in regulation of gene expression between NPSR1-A and NPSR1-B could be seen in other cell types, we transiently transfected two additional cell lines, human epithelial lung carcinom (A549) and human neuroblastoma (SH-SY5Y). The same eleven genes selected for the concentration/time-response study were analysed (*CGA*, *CCL20*, *IL8, PCK1*, *SERPINB2, AREG*, *NTS*, *NR4A2*, *EGR1*, *FOS*, and *CD69*). Although most genes differentially expressed in the HEK-293 cells were altered in these cell lines as well (no expression was however detected for *NR4A2*, *SERPINB2 *or *AREG *in SH-SY5Y cells and *SERPINB2 *in A549 cells), the effect of NPS stimulation on relative gene expression was weaker compared to NPSR1-A or -B overexpressing HEK-293 cells. However, similarly to HEK-293 cells, genes regulated by NPSR1-B were consistently less altered compared to NPSR1-A in both cell lines (Figure 4a, b).

### cAMP and Ca^2+ ^assays

**Figure 5:**
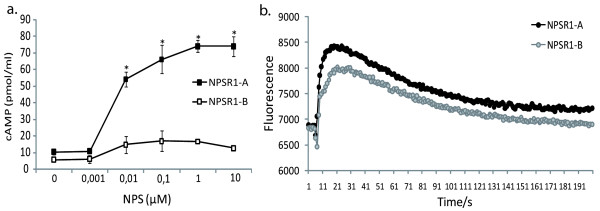
**cAMP and Ca^2+ ^response downstream NPS-NPSR1-A and -B signalling**. Intracellular second messenger response was measured on transiently NPSR1-A or -B overexpressing HEK293 cells stimulated with NPS. The cAMP response (a) was measured in a concentration-response way showing a greater increase of cAMP in NPS-NPSR1-A stimulated cells. In the Ca^2+ ^experiment (b) a fixed dose of 1μM NPS was used and intracellular Ca^2+ ^accumulation was measured before and after injection (t = 5 s) of NPS. The Ca^2+ ^also illustrates an increased response in the NPSR1-A overexpressing cells compared to NPSR1-B. cAMP data is shown as mean ± SEM and * indicates significant (p < 0.05) difference between NPSR1-A and -B. The Ca^2+ ^plot illustrates data from a representative experiment.

Downstream response of cAMP and Ca^2+ ^was measured before and after NPS stimulation of NPSR1-A and -B transiently overexpressing HEK-293 cells. The concentration-response curve for the cAMP assay illustrated that both -A and -B uses cAMP as a second messenger but the response generated by NPSR1-A was much stronger than -B (Figure 5a). The EC_50_-values for NPS-induced cAMP formation for both NPSR1-A and NPSR1-B were close to 5 nM, respectively (Table 2). In the Ca^2+ ^experiment we looked at the release of Ca^2+^, before and after injection of 1 μM NPS to the cells. Here we could also see that the increase of intracellular Ca^2+^, in response to NPS stimulation, was higher in NPSR1-A than in -B (Figure 5b).

### Reporter assays

**Figure 6:**
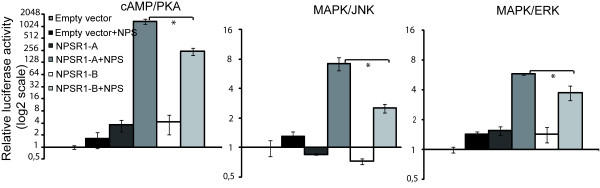
**Reporter assays on NPS-NPSR1 isoform specific activation of cAMP/PKA, MAPK/JNK and MAPK/ERK pathways**. NPS stimulated NPSR1-A or -B overexpressing HEK-293 cells were assayed for transcription factor complex activation of pathways related to GPCR signalling. The results demonstrate that NPSR1-A is a more efficient activator, with a more than 6 fold change for the cAMP/PKA pathway. Data shown as means relative to an empty vector control ± SEM. * Indicates significant (p < 0.05) difference between NPSR1-A and -B.

To further investigate differences seen in gene regulation between NPSR1-A and NPSR1-B, we studied activation of transcription factors representing three signalling pathways; cAMP/PKA, MAPK/JNK and MAPK/ERK. In these experiments, HEK-293 cells were transiently transfected with NPSR1-A, -B or empty pCMV vector together with an inducible transcription factor responsive luciferase construct. Cells were stimulated with NPS (1 μM) for 6 h and assayed for the relative luciferase activity. We observed that NPSR1-A induced a stronger response than NPSR1-B of the cAMP/PKA pathway (six fold) and one and a half (ERK) to two fold stronger (JNK) activator of the MAPK pathways (Figure 6). The results further supported the expression-array experiment and illustrated that NPSR1-A yields a stronger activation of transcription factor complexes.

### Phosphorylation site-directed mutagenesis

**Figure 7:**
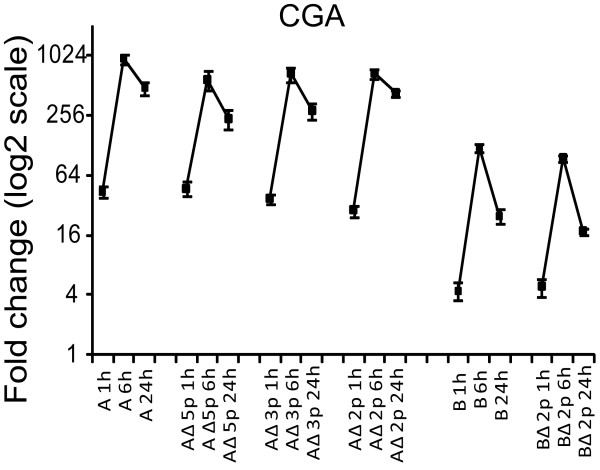
**Phosphorylation site-directed mutagenesis of NPSR1-A and NPSR1-B C-termini**. Relative *CGA *mRNA expression after 1, 6 and 24 h NPS stimulation of HEK-293 cells overexpressing NPSR1-A or -B containing phosphorylation site mutations in the C-termini. From left to right: NPSR1-A,-AΔ5p, -AΔ3p, -AΔ2p, NPSRI-B and -BΔ2p. Expression shown as means relative to an NPS stimulated empty vector control ± SEM.

To dissect the mechanisms of different regulatory effects between the NPSR1-A and -B isoforms, we performed phosphorylation site-directed mutagenesis. The NPSR1-A and -B isoforms possess distinct C-termini which, together with the third intracellular loop, contain phosphorylation sites potentially important for arrestin docking [18]. Phosphorylation occurs predominantly on serine (S) and threonine (T) residues [32] and, as illustrated in Figure 1b, NPSR1-A carries five unique C-terminal phosphorylation sites, whereas NPSR1-B only two. We generated NPSR1-A constructs with all five unique phosphorylation sites in the C-terminal mutated to alanine (AΔ5p), three sites mutated (AΔ3p) and two sites mutated (AΔ2p) and NPSR1-B constructs with the only two unique sites mutated (BΔ2p) (Additional file 2, Figure S1). HEK-293 cells were transiently transfected with the mutant constructs or the wild type pCMV-NPSR1-A or NPSR1-B, stimulated with NPS for 6 h and the downstream expression of a subset of genes were measured with qRT-PCR. The results showed that the removal of phosphorylation sites did not have any effect on downstream gene expression (representative gene shown in Figure 7), suggesting that the significant differential effect between NPSR1-A and -B involves mechanism(s) distinct from simple phosphorylation differences.

## Discussion

The increasing number of positive association studies supports the concept that *NPSR1 *is mechanistically involved in asthma and allergy [1-8,33-35], and also in other diseases [9-12,36]. It is therefore highly important to understand its functions in more detail. We have previously identified downstream target genes for NPS-NPSR1-A signalling using stably NPSR1-A overexpressing HEK-293 cells, and found that many of the up regulated genes are involved in cellular processes such as cell proliferation, morphogenesis and immune responses [27]. Furthermore, we have observed that out of seven splice variants only the full-length variants NPSR1-A and NPSR1-B are transported to the plasma-membrane in transiently transfected COS-1 cells [16], which we can in this study show for NPSR1-A and -B in transiently transfected HEK-293 cells as well. The two NPSR1 isoforms differ in the C-terminal where the last 29 amino acids are unique for NPSR1-A, and the last 35 for NPSR1-B (Figure 1b). Although there is low endogenous expression of GPCRs in most native tissues and cells, generally, NPSR1-A is expressed at a higher level when compared with NPSR1-B and has a more ubiquitous expression pattern. However, in some inflammatory conditions, both isoforms are up regulated and in some cases, e.g., in smooth muscle cells and epithelial cells in asthmatic airways, NPSR1-B may be even more abundant than -A [1,9,10]. In inflammatory cells, such as neutrophils, monocytes and eosinophils, both NPSR1-A and -B are up regulated, but NPSR1-B is more abundant in, e.g., CD4+ T cells [9,19]. Monocytic cell lines stimulated with pro-inflammatory stimuli, such as TNFα and IFNγ, generated elevated *NPSR1 *mRNA levels [10]. These data not only point towards an important role for NPSR1 in inflammation but also to more specific and perhaps distinct roles for NPSR1-A and -B.

Since functional differences and properties of NPSR1-B have not yet been clearly examined our primary aim was to investigate this, using a cell line, HEK-293, that would make the results interpretable in the background of previous relevant studies on NPSR1. When using genome-scale expression arrays to investigate downstream target genes of NPS stimulated HEK-293 cells, transiently transfected with NPSR1-A or NPSR1-B, we found that target gene expression was regulated in an isoform-specific manner. We found the same set of genes to be induced, but NPSR1-A almost exclusively yielded a stronger induction than NPSR1-B, despite equal expression of receptor protein on the plasma membrane (Figure 2a, c). Out of the NPSR1-A induced genes with a more than two fold differential expression, half displayed no significant induction by the NPSR1-B isoform. However, these genes were frequently found to be regulated by the B isoform as well, when looking at genes less than two fold differentially expressed. Of the genes up regulated more than two fold, by both NPSR1-A and -B, a striking majority showed stronger induction by -A. Most of the genes mediated by the NPSR1-A isoform were the same as in our previous expression-array experiment, using NPS stimulated stably NPSR1-A overexpressing HEK-293 cells [27]. However, some new downstream target genes were identified in the present study. These include *CCL20*, *CD69 *and *PTX3*.

Since genes can be early or late responder genes, and our expression-array data only show a cross-section of genes expressed at six hours, we investigated if NPSR1-A and -B required different duration of NPS stimulation to activate gene expression. In the same manner, we also tested if the concentration-response effect differed between the isoforms. The results showed that NPS-NPSR1 regulated gene expression in a time- and concentration-dependent manner and that NPSR1-A, even though the EC_50_-values were similar between the isoforms, was overall a stronger inducer of gene expression. This supports the fact of a clear difference in signalling properties between NPSR1-A and -B. Out of the 11 genes investigated by qRT-PCR, we only found one gene specifically regulated by NPSR1-A, Neurotensin (*NTS*), and one gene showing greater induction by NPSR1-B, CD69 antigen (*CD69*) (Figure 3). *NTS *is a neuropeptide widely distributed throughout the central nervous system and it has been linked both to regulation of circadian rhythms [37] and IBD-related oncogenesis [38]. *CD69 *is an early activation marker of regulatory T cells (Tregs). Tregs are important in monitoring the balance between T-helper type 2 (T_H_2) cells and T_H_1 cells, important for asthma and other inflammatory disorders [39]. These observations connect well to the genetic associations reported for NPSR1. Recently, CD69 was reported to specifically control the pathogenesis of allergic airway inflammation [40], and over expression of the NPSR1-B isoform in asthmatic airways [1] might thus drive this pathway toward allergic inflammation.

The majority of protein-coding genes in the human genome are known to undergo alternative splicing [41] but it is still not fully understood how the cell- and tissue-specific splicing is regulated [42]. Expression profiling of NPSR1-A and NPSR1-B demonstrates that the isoforms are differentially expressed in tissues and cells, suggesting tissue-specific regulation of alternative splicing [1,9,16,19]. Hence, we investigated NPS-NPSR1-A and -B signalling in lung cells (A549) and neuronal cells (SH-SY5Y). We identified some differences between cell lines in terms of which genes are regulated which might be due to different gene regulatory mechanisms that are active in different cell types. However, we conclude that although the induction of these selected genes is not of the same magnitude as in HEK-293 cells, NPSR1-A is the stronger activator also in A549 and SH-SY5Ycells. In these cell lines we could not find specific genes regulated by one isoform alone.

To further study NPS-NPSR1 isoform-specific downstream activity, we investigated intracellular cAMP accumulation and Ca^2+ ^release from each respective isoform. The response generated by NPSR1-A was clearly higher for both second messengers. We also looked at activation of downstream transcription factor complexes. Upon activation of GPCRs the receptor signals both through the traditional G-proteins, but also through alternative pathways such as mitogen-activated protein kinases (MAPKs) [18]. The results illustrated that both NPSR1-A and -B induced transcription factors representing cAMP/PKA and MAPK pathways, but that signalling through NPSR1-A gave a stronger response. These data confirmed our expression-array findings and suggested that the difference in signalling is already seen at a second-messenger and transcription factor level.

Upon stimulation of GPCRs, conformational changes of the receptor activate G proteins. Recent studies have shown that the C-terminal of the receptor is most likely not involved in the activation, but rather regulates the selectivity towards which G protein is utilized [43]. Previous studies demonstrate that NPS-NPSR1-A signals through Gαq and Gαs [14,15] and since activation of NPSR1-A and -B induces the same genes, it is likely that they select the same type of G-proteins. After activation, the GPCRs are rapidly desensitized by phosphorylation and arrestin binding. These events predominantly occur on serine (S) and threonine (T) residues within the C-terminal and the third intracellular loop [32]. After desensitization, the receptor is internalized and targeted for degradation, redirected signalling through G-protein independent pathways (e.g. MAPK) or recycled back to the membrane [18]. Previous studies have shown that phosphorylation site-directed mutations at the C-terminal of GPCRs severely impaired both the ability to undergo phosphorylation and to recruit arrestin [44,45]. It has also been demonstrated that conformational changes that improve C-terminal phosphorylation also enhances arrestin binding and endocytosis [46]. As depicted in Figure 1b, NPSR1-A contains more than twice as many unique phosphorylation sites as NPSR1-B. This opens up for the possibility that the -A isoform undergoes a faster turnover and hence is able to affect downstream gene expression in a more efficient way. To test this hypothesis we performed phosphorylation site-directed mutagenesis on both NPSR1-A and -B, replacing all or a subset of potential phosphorylation sites with alanine. The results revealed however that the number of phosphorylation sites in the NPSR1-A and NPSR1-B C-terminal did not seem to affect the difference in downstream gene regulation observed between the isoforms, even though minor effects were seen within each isoform group. The mechanisms behind isoform specific gene regulation will need further investigation.

## Conclusions

Several studies point towards an important role for NPSR1 in asthma and allergy, and moreover an explicit role for the -A and -B isoforms. Prior studies have not revealed any significantly altered second messenger response between NPSR1-A and NPSR1-B or investigated differences in downstream gene expression [16,23]. From this study we conclude that signalling of NPS-NPSR1-A and -B affect the same pathways but in an isoform specific manner, identifying NPSR1-A as a receptor with stronger signalling effects with few, but possibly important exceptions (such as *CD69*). In addition, we examined unique phosphorylation sites in the C-termini as a plausible explanation for discrepancy, but our data indicated that the differential effect was more complicated than a simple phosphorylation target difference. Our results suggest an isoform-specific link to pathogenetic processes in allergic airways.

## Competing interests

The authors declare that they have no competing interests.

## Authors' contributions

COP, JV, MDA, JK conceived and designed the experiments. COP, JV, FA, CS performed the experiments. COP, JV, SB, MA, MDA, CS analyzed the data. LS, VP, HA, JK contributed reagents/materials/analysis tools. COP wrote the paper. JV, CS, JK revised the manuscript for important intellectual content. All authors read and approved the final manuscript.

## Pre-publication history

The pre-publication history for this paper can be accessed here:

http://www.biomedcentral.com/1471-2466/11/39/prepub

## Supplementary Material

Additional file 1 **Table S1. Primers sequences**. Sequences for all primers used for qRT-PCR. Click here for file

Additional file 2 **Figure S1. Schematic picture of the C-terminal tail of NPSR1-A and -B illustrating the phosphorylation site-directed mutagenesis constructs**. Three different constructs were designed for NPSR1-A, AΔ5p, AΔ3p, AΔ2p, and one for NPSR1-B; BΔ2p exchanging serine (S) or threonine (T) sites for alanine (A). Click here for file

Additional file 3 **Figure S2. NPS-NPSR1 concentration- and time-response**. Relative expression monitored by qRT-PCR downstream NPSR1-A or -B overexpressing HEK-293 cells for NPS dose- (6 h stimulation) and time-response (2μM NPS), for eight representative genes. Data shown as relative to an NPS stimulated empty vector control. * Indicates at which concentrations or time points the difference between NPSR1-A and -B was significant (p ≤ 0.05). Click here for file
