# Supplementary material for: The asthma candidate gene NPSR1 mediates isoform specific downstream signalling
Source: BMC Pulm Med. 2011 Jun 27;11:39. doi: 10.1186/1471-2466-11-39 (PMC3142248; doi:10.1186/1471-2466-11-39)
Supplement: Additional file 1 — Table S1. Primers sequences. Sequences for all primers used for qRT-PCR. [file 1471-2466-11-39-S1.PDF]

Additional file 1.
Table S1

Gene name	**Forward**	**Reverse**
NPSR1	CATTCTTGCCTTCATCTGCTGTT	CTCCTGGGTGTCTGGAAGGA
NPSR1-A	CCTGCAGGGAGCAAAGATCA	AATCTGCATCTCATGCCTCTCA
NPSR1-B	CCCCCTCATCTACTGTGTCTTCA	TCGTTGAGGGCAGAGCATTA
CGA	TCCCACTCCACTAAGGTCCAA	CCCCATTACTGTGACCCTGTT
CCL20	GCGAATCAGAAGCAGCAAG	GATTTGCGCACACAGACAAC
IL-8	TGACTTCCAAGCTGGCCGTGGCT	TCTCAGCCCTCTTCAAAAACTTCTC
PCK1	CTGTGGATCTCCCTTCGAGA	GATCAGGCTGACACAGCTCA
NR4A2	GAACTGTTTGTCCTTCGATTAGCA	CCCATTGCAAAAGATGAGTTTACC
SERPINB2	GATGGCCAAGGTGCTTCA	TGCAAAATCGCATCAGGATA
AREG	GTAACATGCAAATGTCAGCAAGA	TTTCGTTCCTCAGCTTCTCC
EGR1	CACCTGACCGCAGAGTCTT	AGCGGCCAGTATAGGTGATG
FOS	AGGTGGAACAGTTATCTCCAGAA	CTTCTCCTTCAGCAGGTTGG
NTS	CTGTGCTCAGATTCAGAAGAGG	CTCAGCTGGGCTGTTCAAAT
CD69	AGAAAATGATGCCACCAGTCC	AGCATGAAGAAACATGGCTGT
HPRT	TCAGGCAGTATAATCCAAAGATGGT	AGTCTGGCTTATATCCAACACTTCG
GAPDH	ACGGATTTGGTCGTATTGGG	TGATTTTGGAGGGATCTCGC
NPSR1-A Δ2P*	TGGCGTTCCGGGAGAGAGCTGAGA GGCATGAGATGCAGATTCTGGCCAA GCCAG	CTGGCTTGGCCAGAATCTGCATCTCATG CCTCTCAGCTCTCTCCCGGAACGCCA
NPSR1-A Δ2p*	TGACGTTCCGGGAGAGAGCTGAGA GGCATGAGATGCAGGTTCTGGCCAA GCCAG	CTGGCTTGGCCAGAATCTGCATCTCATG CCTCTCAGCTCTCTCCCGGAACGTCA
NPSR1-A Δ3p	TGCAGGGAGCAAAGAGCACAGGAT	CCGGAACGCCATTCTGGCATCCTGTGCT
NPSR1-B Δ2p	AACTGGAAGGGTGCTTGGCCAGGTG TACCTGCCTGGGC	GCCCAGGCAGGTACACCTGGCCAAGCAC CCTTCCAGTT

* Two different -A Δ2p/P primers were used since -A Δ2P was designed to change the last S and T residues in the already generated NPSR1-A Δ3p constructs, resulting in a NPSR1-A Δ5p construct.
