# Supplementary figures and images for: The asthma candidate gene NPSR1 mediates isoform specific downstream signalling
Source: BMC Pulm Med. 2011 Jun 27;11:39. doi: 10.1186/1471-2466-11-39 (PMC3142248; doi:10.1186/1471-2466-11-39)

Additional file 2.

Figure S1

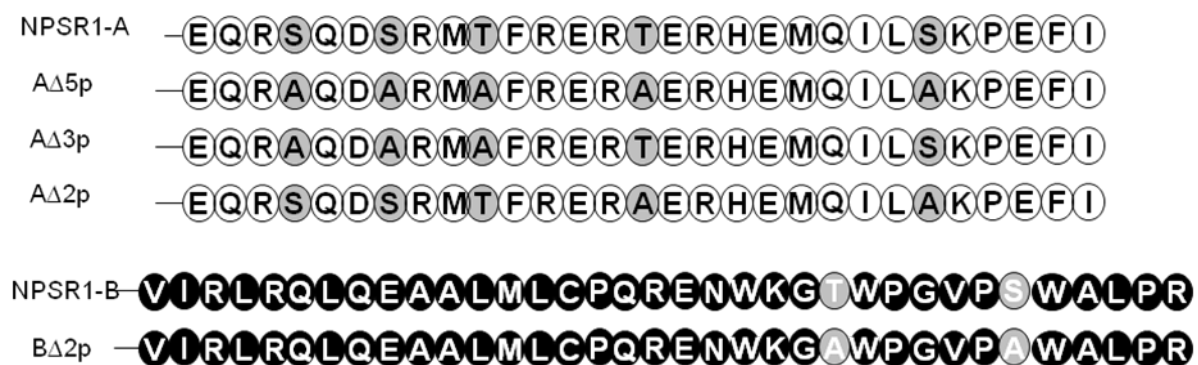

Supplement: Additional file 2 — Figure S1. Schematic picture of the C-terminal tail of NPSR1-A and -B illustrating the phosphorylation site-directed mutagenesis constructs. Three different constructs were designed for NPSR1-A, AΔ5p, AΔ3p, AΔ2p, and one for NPSR1-B; BΔ2p exchanging serine (S) or threonine (T) sites for alanine (A). [file 1471-2466-11-39-S2.PDF]

**Additional file 3**  
**Figure S2**

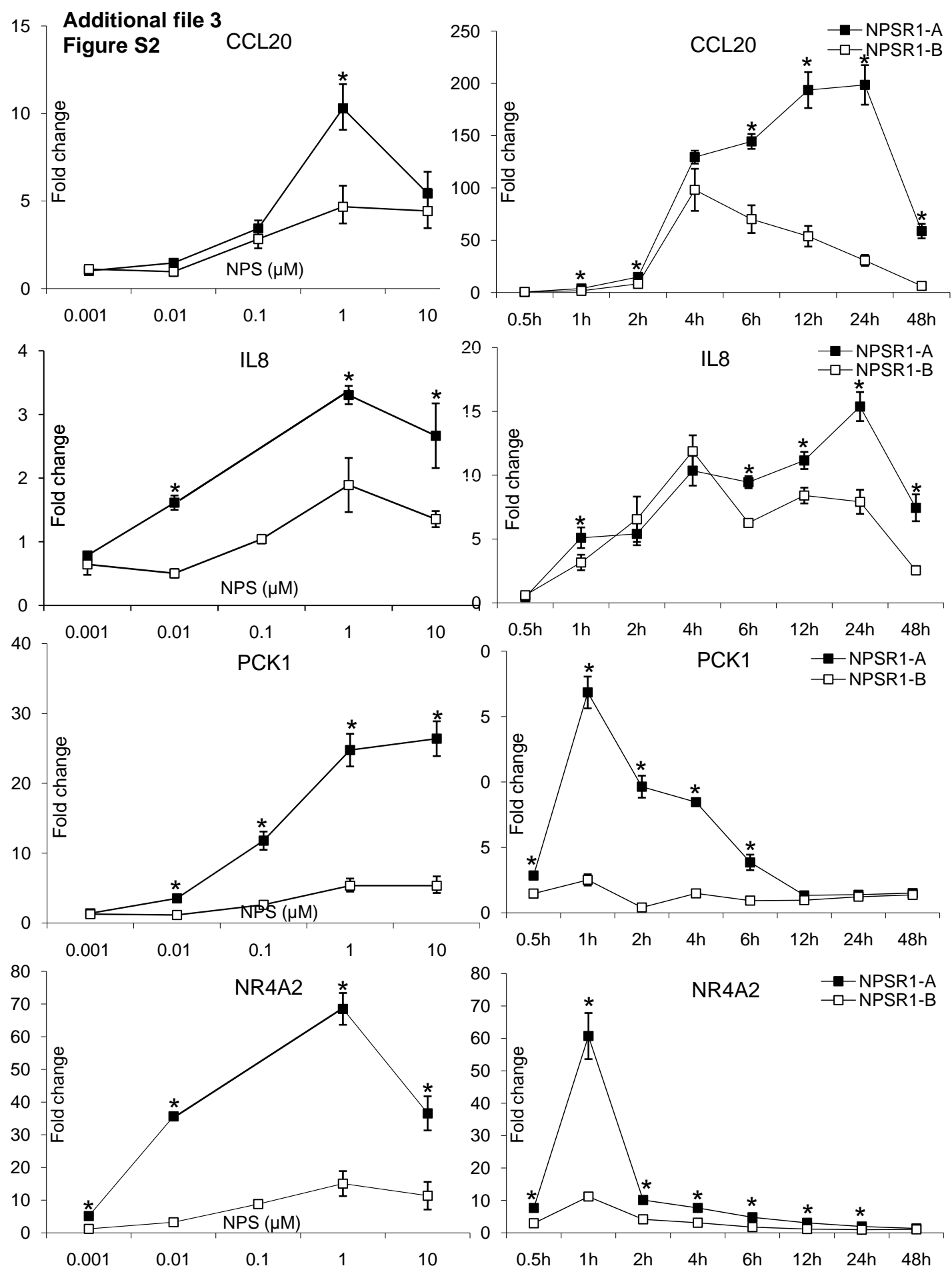

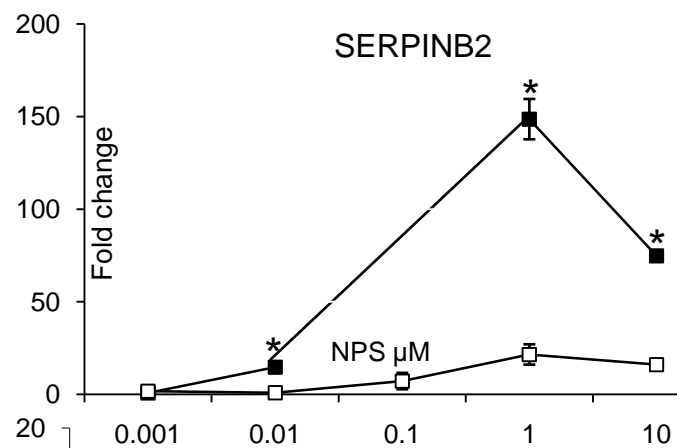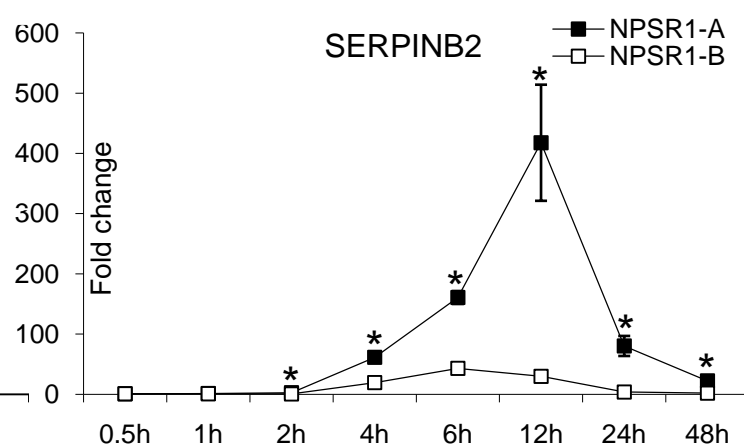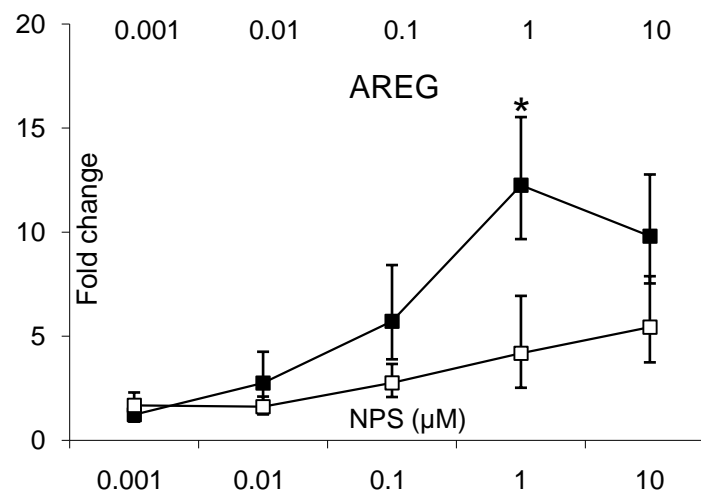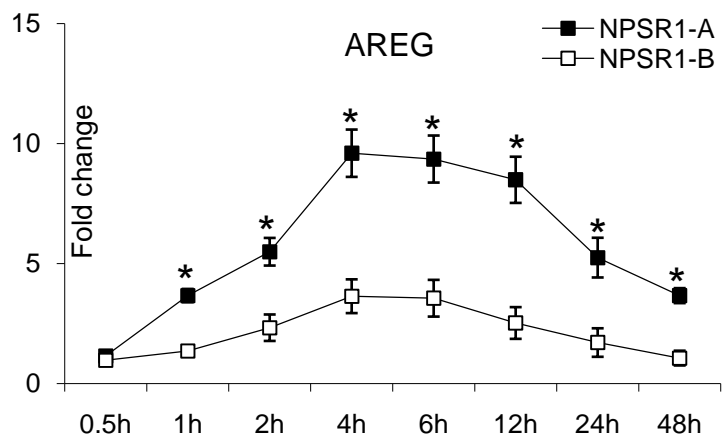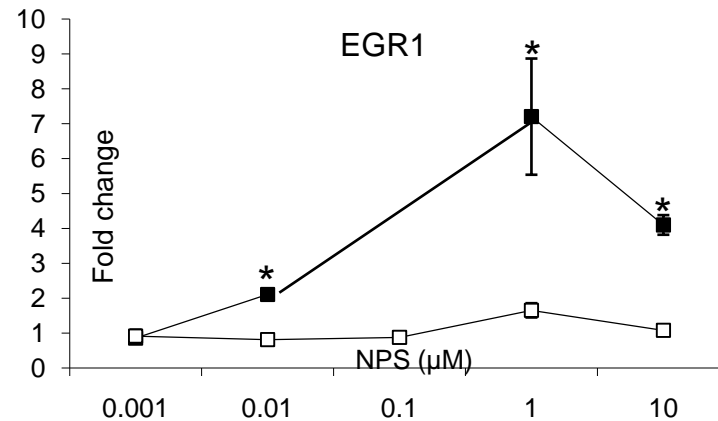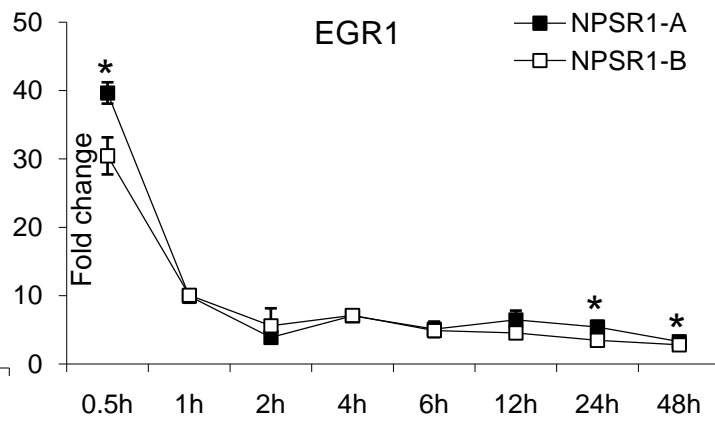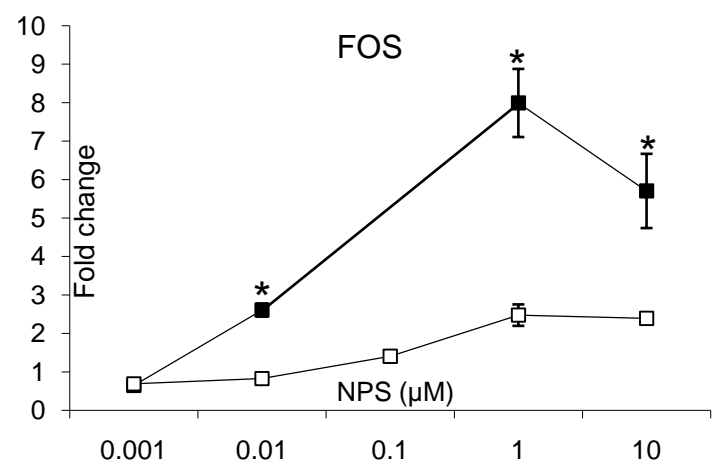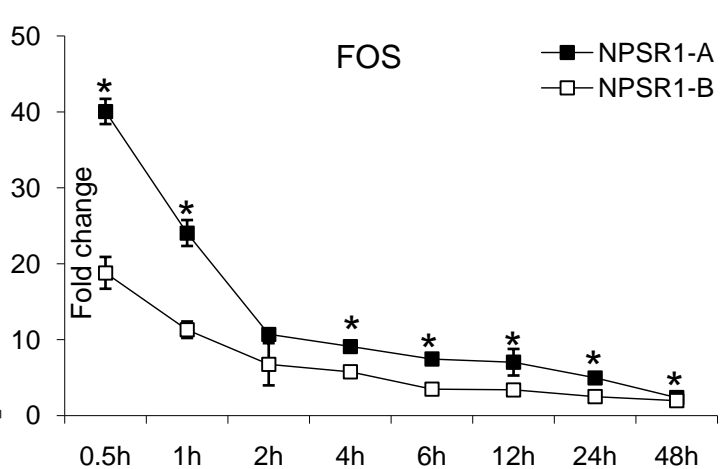

Supplement: Additional file 3 — Figure S2. NPS-NPSR1 concentration- and time-response. Relative expression monitored by qRT-PCR downstream NPSR1-A or -B overexpressing HEK-293 cells for NPS dose- (6 h stimulation) and time-response (2μM NPS), for eight representative genes. Data shown as relative to an NPS stimulated empty vector control. * Indicates at which concentrations or time points the difference between NPSR1-A and -B was significant (p ≤ 0.05). [file 1471-2466-11-39-S3.PDF]
